# Single‐Incision Anterolateral Thoracotomy for LVAD Implantation: A Novel Minimally Invasive Approach

**DOI:** 10.1111/aor.70070

**Published:** 2025-12-01

**Authors:** Ali Saad Merzah, Günes Dogan, Alina Zubarevich, Stefan Rümke, Bastian Schmack, Ezin Deniz, Jasmin Hanke, Arjang Ruhparwar, Jan D. Schmitto, Alexander Weymann

**Affiliations:** ^1^ Department of Cardiac‐, Thoracic‐, Transplantation and Vascular Surgery Hannover Medical School Hannover Germany

**Keywords:** advanced heart failure, anterolateral thoracotomy, HeartMate 3, LVAD, mechanical circulatory support, minimally invasive surgery, single incision

## Abstract

A novel single‐incision anterolateral thoracotomy technique enables complete HeartMate 3 implantation without sternotomy, minimizing surgical trauma and accelerating recovery. This minimally invasive approach is feasible, safe, and may represent a new standard in advanced heart failure surgery.

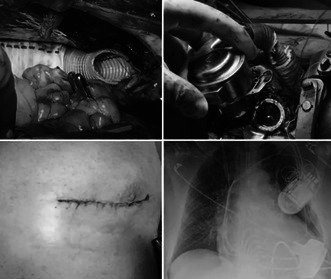

## 1 Introduction

The prevalence of advanced‐stage heart failure has led to a significant increase in the utilization of left ventricular assist devices (LVADs) worldwide. Technological advancements and growing surgical expertise have improved patient outcomes, reducing complications and enhancing device performance. The HeartMate 3 (Abbott, Chicago, IL, USA) represents the latest generation of LVADs, featuring a fully magnetically levitated rotor that minimizes shear stress and optimizes hemocompatibility [1, 2].

Despite these advancements, traditional LVAD implantation via full sternotomy remains invasive, associated with substantial surgical trauma and prolonged recovery times [3, 4]. Minimally invasive techniques for LVAD implantation have been evolving rapidly, driven by the need to reduce surgical trauma and improve patient outcomes. Approaches such as upper hemi‐sternotomy combined with anterior lateral thoracotomy have been widely adopted as alternatives to traditional full sternotomy. These techniques maintain chest wall integrity, minimize postoperative bleeding, and reduce the risk of right heart failure by preserving pericardial structure. Additionally, lateral thoracotomy has been utilized for specific cases, allowing access to the left ventricular apex without compromising the entire sternum [5, 6].

These established approaches have paved the way for further innovations, such as the single‐incision anterolateral thoracotomy described in this report, which represents a significant step forward in minimizing surgical invasiveness while maintaining procedural efficacy.

## 2 Case Presentation

A 60‐year‐old male patient presented with terminal heart failure (NYHA class IV, Intermacs profile 3) secondary to ischemic cardiomyopathy. The patient had a history of coronary artery disease with multiple interventions, including stent placements, and refractory ventricular tachyarrhythmias. Preoperative transthoracic echocardiography revealed severe left ventricular dysfunction (ejection fraction ~10%) and moderate mitral regurgitation.

Standard LVAD evaluation, including left heart catheterization and pulmonary function tests, confirmed the need for mechanical circulatory support. The patient exhibited significant functional limitation, with a walking distance of less than 100 m, necessitating urgent intervention.

In addition, a preoperative contrast‐enhanced CT scan of the chest was performed to evaluate the ascending aorta and thoracic anatomy. The scan demonstrated a non‐calcified, non‐atheromatous ascending aorta, which we considered a prerequisite for performing the aortic outflow anastomosis through a single left anterolateral thoracotomy without additional access.

## 3 Surgical Technique

The operation was performed under general anesthesia with the patient in a supine position and the left hemithorax slightly elevated. Cardiopulmonary bypass was established via femoral arterial and venous cannulation.

A single left anterolateral thoracotomy measuring approximately 8–10 cm was performed in the fifth intercostal space, providing exposure of both the left ventricular apex and the ascending aorta through the same incision. The pericardium was opened anterior to the aorta and over the apex, and several stay sutures were placed to improve exposure while maintaining pericardial support for the right ventricle.

To perform the aortic outflow anastomosis, a small ancillary skin incision (< 1 cm) was made in the second intercostal space close to the sternum to introduce a Satinsky side‐biting clamp for partial aortic occlusion. Through this access, the ascending aorta was clamped, and the LVAD outflow graft was anastomosed end‐to‐side to the ascending aorta under direct vision from the main thoracotomy. This sequence—outflow graft first, followed by pump implantation—provides optimal visualization of the aorta before the device occupies the limited intrathoracic space.

**FIGURE 1:**
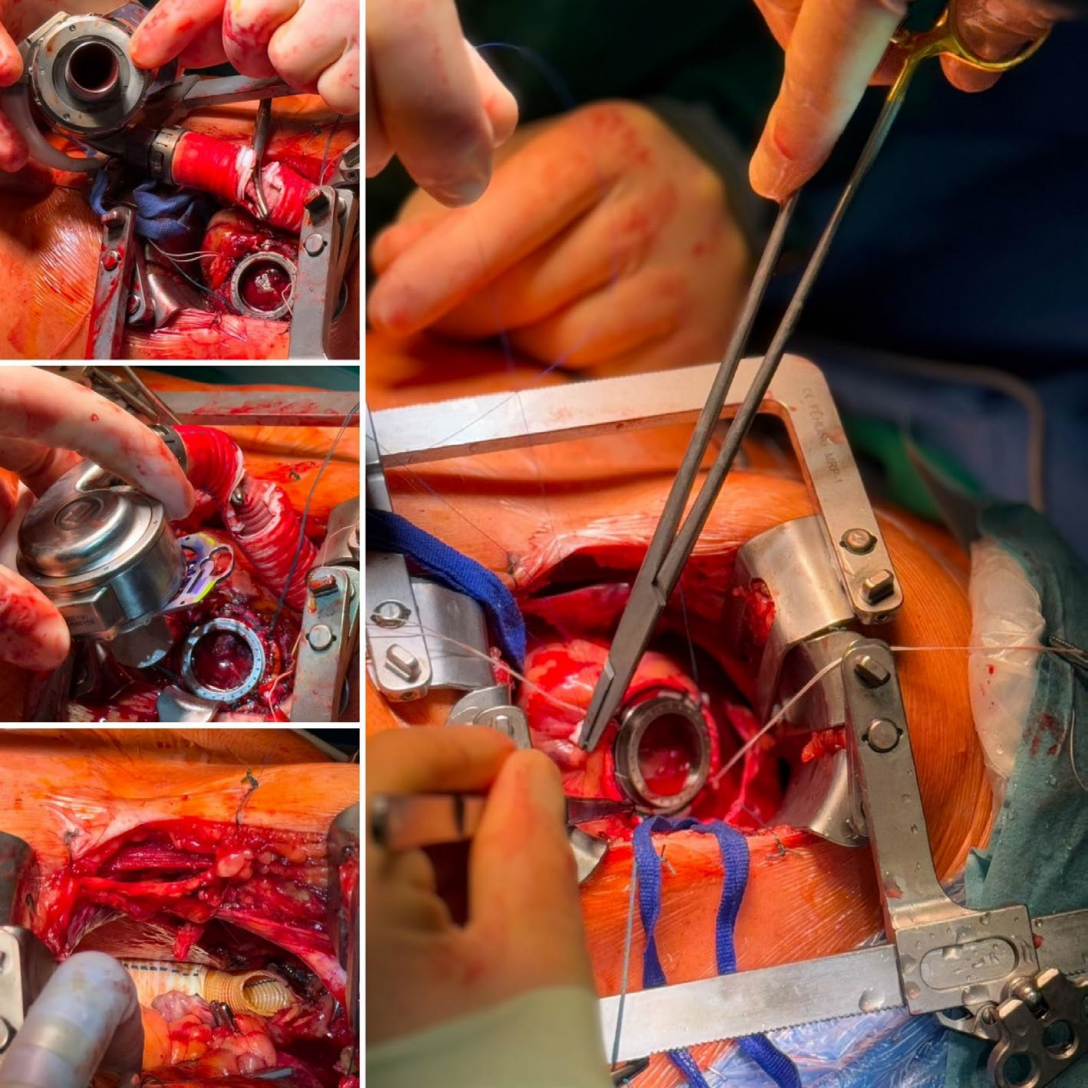
Intraoperative view through the single left anterolateral thoracotomy demonstrating exposure of the left ventricular apex and the ascending aorta. The outflow graft is shown being routed toward the ascending aorta for end‐to‐side anastomosis. Note the preserved pericardium around the right ventricle and the absence of any additional thoracic access. [Color figure can be viewed at wileyonlinelibrary.com]

After completion of the outflow anastomosis and graft routing without tension or kinking, the left ventricular apex was cored and the pump was implanted in standard fashion. Continuous CO_2_ insufflation was used throughout the procedure. De‐airing was performed step‐by‐step: the outflow graft was filled and vented before final tightening, the ventricle was gently manipulated, and intraoperative transesophageal echocardiography (TEE) was used to confirm complete air evacuation before initiating full LVAD support. Thoracic drains were placed, and the thoracotomy was closed in layers with meticulous hemostasis (Figure 1).

**FIGURE 2:**
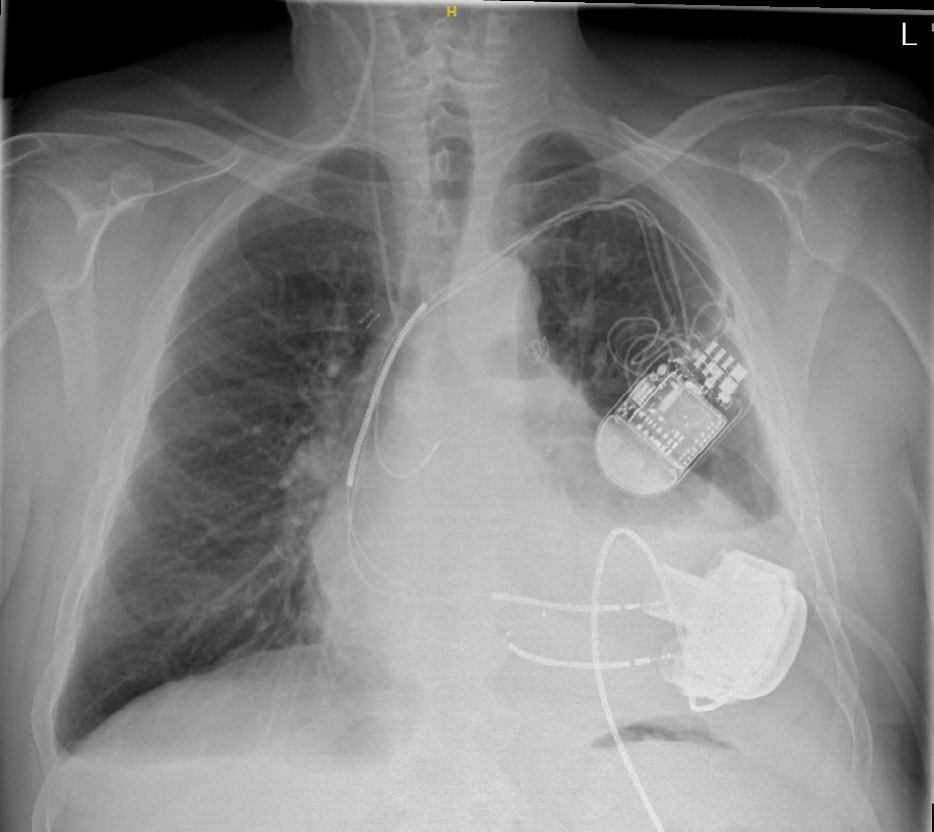
Postoperative chest X‐ray showing the correct position of the HeartMate 3 device. The preserved sternum and intact right hemithorax confirm the single‐incision approach. [Color figure can be viewed at wileyonlinelibrary.com]

The surgery lasted 2 h and 33 min, with a bypass duration of 110 min. No complications were encountered during the procedure, and meticulous hemostasis was achieved. Postoperatively, thoracic drains were placed for fluid management (Figure 2).

## 4 Postoperative Course

The patient exhibited a remarkable recovery. He was extubated within 7 h, 28 min postoperatively and transitioned to oral anticoagulation without complications. No device‐related or surgical issues, such as bleeding, arrhythmias, or infections, were observed. By the 1st postoperative day, the patient was transferred to the Intermediate care unit (IMC) and by the 5th postoperative day to a regular normal ward and prepared for discharge in stable condition with optimal LVAD function.

## 5 Discussion

The single‐incision anterolateral thoracotomy approach for LVAD implantation represents a further step in the evolution of minimally invasive LVAD surgery. This technique aims to reduce surgical trauma while maintaining procedural safety and reproducibility. By concentrating all working steps within one principal incision, the approach preserves complete chest‐wall integrity and eliminates the morbidity associated with multiple access sites.

From a surgical perspective, preserving the sternum and the right thoracic wall maintains structural stability, minimizes postoperative discomfort, and facilitates potential future procedures such as re‐entry for heart transplantation or right ventricular assist device (RVAD) implantation. In our daily practice as cardiac surgeons and researchers, we also recognize a distinct psychological component: many patients express strong anxiety toward sternotomy, associating it with pain, scarring, and prolonged recovery. When presented with a sternum‐sparing option, they consistently demonstrate greater willingness and confidence to undergo surgery. Thus, this single‐incision approach not only advances surgical minimalism but also aligns with patient expectations and emotional comfort.

Compared with other sternal‐sparing LVAD strategies—such as upper hemi‐sternotomy combined with a right anterior thoracotomy or dual mini‐thoracotomies—our approach provides a direct and safe route to both the left ventricular apex and the ascending aorta through one incision. The potential benefits include reduced operative time, limited bleeding, and faster mobilization while maintaining optimal exposure for safe anastomosis. The small ancillary incision (< 1 cm) for Satinsky clamp insertion allows partial aortic occlusion without compromising the single‐incision concept.

Nevertheless, careful preoperative imaging is mandatory to rule out calcified or atheromatous aortic segments that might complicate clamping or suturing. In the case of posterior anastomotic bleeding after pump initiation, our safety strategy includes temporary reduction of LVAD speed or partial CPB return, gentle elevation of the aorta using pericardial stay sutures, and, if necessary, controlled extension of the existing thoracotomy rather than creating a new access.

While this single‐incision technique cannot replace all established minimally invasive routes, it broadens the spectrum of tailored options for LVAD implantation. Larger patient series and long‐term follow‐up will be required to confirm the reproducibility, safety, and potential recovery benefits suggested by this initial experience.

## 6 Conclusion

This case highlights the feasibility and benefits of a single‐incision anterolateral thoracotomy for LVAD implantation. The technique minimizes surgical trauma, accelerates recovery, and maintains excellent hemodynamic support. Further studies are warranted to validate these findings and establish this approach as a standard in advanced heart failure therapy.

## Author Contributions

**Ali Saad Merzah :** conceived the case report, contributed to the surgical planning and procedure, collected the clinical data, performed the literature review, and drafted the manuscript. **Günes Dogan:** contributed to surgical planning, intraoperative management, data interpretation, and critical revision of the manuscript. **Alina Zubarevich, Stefan Rümke, Bastian Schmack, Ezin Deniz, and Jasmin Hanke:** contributed to perioperative patient management, data collection, and critical review of the manuscript. **Arjang Ruhparwar and Jan D. Schmitto:** contributed to the development of the minimally invasive surgical concept, supervised the clinical management, and critically revised the manuscript for important intellectual content. **Alexander Weymann:** supervised the project, contributed to data interpretation, and critically revised and approved the final version of the manuscript. All authors read and approved the final version of the manuscript.

## Conflicts of Interest

The authors declare no conflicts of interest.

## Data Availability

The data that support the findings of this study are available from the corresponding author upon reasonable request.
